# Influence of Litter Diversity on Dissolved Organic Matter Release and Soil Carbon Formation in a Mixed Beech Forest

**DOI:** 10.1371/journal.pone.0114040

**Published:** 2014-12-08

**Authors:** Andrea Scheibe, Gerd Gleixner

**Affiliations:** Max Planck Institute for Biogeochemistry, Jena, Germany; University of Oxford, United Kingdom

## Abstract

We investigated the effect of leaf litter on below ground carbon export and soil carbon formation in order to understand how litter diversity affects carbon cycling in forest ecosystems. ^13^C labeled and unlabeled leaf litter of beech (*Fagus sylvatica*) and ash (*Fraxinus excelsior*), characterized by low and high decomposability, were used in a litter exchange experiment in the Hainich National Park (Thuringia, Germany). Litter was added in pure and mixed treatments with either beech or ash labeled with ^13^C. We collected soil water in 5 cm mineral soil depth below each treatment biweekly and determined dissolved organic carbon (DOC), δ^13^C values and anion contents. In addition, we measured carbon concentrations and δ^13^C values in the organic and mineral soil (collected in 1 cm increments) up to 5 cm soil depth at the end of the experiment. Litter-derived C contributes less than 1% to dissolved organic matter (DOM) collected in 5 cm mineral soil depth. Better decomposable ash litter released significantly more (0.50±0.17%) litter carbon than beech litter (0.17±0.07%). All soil layers held in total around 30% of litter-derived carbon, indicating the large retention potential of litter-derived C in the top soil. Interestingly, in mixed (ash and beech litter) treatments we did not find a higher contribution of better decomposable ash-derived carbon in DOM, O horizon or mineral soil. This suggest that the known selective decomposition of better decomposable litter by soil fauna has no or only minor effects on the release and formation of litter-derived DOM and soil organic matter. Overall our experiment showed that 1) litter-derived carbon is of low importance for dissolved organic carbon release and 2) litter of higher decomposability is faster decomposed, but litter diversity does not influence the carbon flow.

## Introduction

Leaf litter decomposition is a fundamental process for nutrient and carbon cycling in forest ecosystems. Litter decomposition results in release of dissolved organic matter (DOM) and thus contributes significantly to carbon and nutrient transfer and storage in soils as well as to the export of carbon with surface or groundwater to the ocean [1], [2], [3], [4]. However, we have little knowledge about the controlling processes that regulate the fluxes and concentrations of DOM in soil solution [5], [6], [7]. Moreover, the contribution of litter-derived carbon to the DOM pool is still under discussion [6], [8], [9], [10], [11].

Under field conditions, DOM shows seasonal changes in concentration, which can be related to litter decomposition processes [3], [12]. Thus, climatic factors like temperature and soil moisture exert an abiotic control on litter decomposition [9], [13], [14]. In addition, biotic factors like litter quality, soil fauna (micro-, meso-, macro-) and the interaction between different litter types influence the decomposition processes [15], [16], [17].

Litter-derived DOM in soil water can be used as an indicator for microbial decomposition [6], [18], [19], [20]. Earlier investigations on litter degradation have shown that litter of high quality with high N, P or low lignin concentrations and consequently low C/N or lignin/N ratios is decomposed faster by the soil microbial community [14], [21], [22], [23]. Litter of higher decomposability is for example produced from European ash (*Fraxinus excelsior* L.), whereas European beech (*Fagus sylvatica* L.) has a lower decomposability [21], [23], [24]. Additionally, the presence of soil fauna (meso- and macro-) can substantially change the decomposition of leaf litter and alter the litter-diversity effects on decomposition [25]. Nevertheless, it is still not well understood, if soil organisms (micro-, meso-, macro-) with a preference of individual litter types in litter mixtures influence the release and transport of litter-derived DOM in the upper soil horizons.

The DOM in soil solutions can originate from fresh organic matter (leaf or root litter, root exudates) or soil organic matter (SOM) [20], [26]. The stable carbon isotope (^13^C) can be used as a tracer to investigate the source and fate of litter-derived carbon into different carbon pools like soil water, the O horizon and mineral soil [26], [27], [28], [29]. Recent improvements coupling high performance liquid chromatography (HPLC) online to isotope ratio mass spectrometry (IRMS) enable fast and reliable DO^13^C measurements in soil water [30], [31], [32]. This has allowed rapid measurements of smaller DOC samples, which opens the possibility to implement long time series investigations.

In this study, we investigated the influence of leaf litter 1) quality and 2) mixture on the export of DOM in soil water and the formation of SOM in the upper soil horizons. Therefore, we established a leaf litter exchange experiment in a deciduous forest in central Germany, using ^13^C labeled litter. We used litter from European ash (*Fraxinus excelsior* L.) and European beech (*Fagus sylvatica* L.), which are the dominant tree species in the Hainich National Park (Thuringia, Germany) and known for their difference in decomposability as described above. Pure and mixed treatments were used to determine a preferred decomposition species and its impact on litter-derived DOM release and SOM production. We hypothesize that the export of carbon from litter decomposition 1) increases with higher leaf litter decomposability and 2) increases in mixed treatments by selective decomposition of ash litter.

## Materials and Methods

### 2.1 Study site

The litter exchange experiment was performed in the Hainich National Park (Thuringia, Germany), which protects the largest closed mixed beech forest in central Germany (∼75 km^2^). The forest grows on a Luvisol developed from loess over Triassic limestone [33], [34]. The soil texture ranged from a silt loam to silt clay loam in the upper (0–30 cm) mineral soil [33]. The mean annual temperature is 7.5°C and the mean annual precipitation is 670 mm (Meteomedia, station Weberstedt/Hainich; 51°06′0″N, 10°31′12″E, 270 m a.s.l.).

The study site (51°06′3.64″N, 10°27′29.93″E) was established in a pure beech stand of the forest near the village Mülverstedt (51°07′0.12″N, 10°30′0″E). To conduct our experiment in the area of the Hainich National Park we had the permission of the National Park administration. The study site of 50 m×50 m was fenced to keep out big game. The soil texture in 0–10 cm was characterized as 3% sand, 82% silt and 15% clay [33]. According to Zanella et al. [35], the forest floor was classified as a dysmull (OL + OF) to hemimoder (OL + OF + discontinuous OH) covering a topsoil (0–5 cm) with pH_KCl_ of 3.3 [36].

### 2.2 Preparation and collection of leaf litter

For the experiment, we used leaf litter from European ash (*Fraxinus excelsior* L.) and European beech (*Fagus sylvatica* L.). Labeled leaf litter was produced in a closed greenhouse with ^13^CO_2_-enriched atmosphere (∼300 ‰ V-PDB) for one growing season [36]. As reference litter (unlabeled treatments), leaf litter of beech and ash were collected in the Hainich National Park. Litter samples were collected in autumn at the beginning of leaf senescence, air dried, carefully mixed and prepared in the lab. Subsamples of all litter types were dried (24 h at 105°C), ground and litter parameters (δ^13^C, organic C, N, C/N and lignin) determined as described in Langenbruch et al. [36]. The labeled and unlabeled litter of ash and beech differed in their initial isotopic signature (δ^13^C) and litter quality parameters (S1 Table).

### 2.3 Experimental design

On the study site, mesocosms (plastic tubes, 20 cm height, Ø 24 cm) were installed on Dec. 8^th^ and 9^th^, 2008. Therefore, intact soil cores (Ø 24 cm; with litter layer, O horizon and mineral soil of 5 cm depth) were transferred into mesocosms and relocated to their original location. The mesocosms were separated from each other by at least 1 m and by at least 2 m from the nearest tree. Each mesocosm contained a mineral soil core of 5 cm depth, the O horizon and the litter layer. Freshly fallen aboveground litter in the mesocosms was removed and replaced by 14.38 g ( = 317.9 g m^−2^) of labeled (^13^C enriched) and/or unlabeled leaf litter of beech and ash on Dec. 12^th^, 2008. The mesocosms were closed at the bottom with 50 µm gauze to exclude root ingrowth. On top, they were covered with fly gauze to prevent external litter input and loss of added litter. Directly underneath the mesocosms porous borosilicate glass suction plates (Ø 12 cm, pore size 1 µm, SPG120-1/8″, UMS GmbH, München, Germany) were installed. A mean suction pressure of 100 hPa was applied biweekly that roughly corresponds to approximately free draining soil water. To calculate the amounts of litter-derived carbon in DOM we applied a correction factor of ∼4 that expanded the area of the glass suction plate (113.1 cm^2^) to the area of the mesocosm (452.4 cm^2^), assuming that in the mean the surface area was coherent to the area of the suction plate.

On the study site, the mesocosms were arranged in three blocks (Fig. S1). Two mesocosms of the following treatments were established at each block: 1) unlabeled beech litter (Be), 2) 1∶1 (m/m) mixture of unlabeled beech and ash litter (BeAs), 3) unlabeled ash litter (As), 4) labeled beech litter (Be*), 5) 1∶1 (m/m) of labeled beech and unlabeled ash litter (Be*As), 6) 1∶1 (m/m) of unlabeled beech and labeled ash litter (BeAs*), 7) labeled ash litter (As*). In total, each block consisted of 14 mesocosms.

### 2.4 Meteorological measurements

Environmental parameters were collected from an Eddy flux tower located in the Weberstedter Holz (51°04′46″N, 10°27′08″E, 440 m a.s.l.) of the Hainich National Park approximately 2.5 km south from the study site [37], [38]. Precipitation (RainGauge, Young, Traverse City, MI, USA) was measured at a forest clearing 800 m away from the tower. Soil temperature was detected with thermistor sensors (PT100, Geraberger Thermometerwerk GmbH, Geschwenda, Germany) at two positions at a soil depth of 5 cm. Soil moisture was measured using Theta-probes (ML-2x, DeltaT, Cambridge, UK) at four positions at a soil depth of 8 cm.

Between two sampling dates the mean precipitation was 31.1 (±20.2 sd) mm (S2 Table, Fig. S2). The soil moisture was 41.0 (±4.5) %, except in the late summer of 2009 where it decreased to a minimum of only 21.3 (±4.7) %. The soil temperature in 5 cm depth showed a clear seasonal pattern with lower temperatures of 0.9 (±1.4)°C during the winter periods (Dec. – Mar.) and a maximum temperature of 17.0 (±0.9)°C on Aug. 09.

### 2.5 Sample collection, preparation and analysis

#### 2.5.1 Soil water sampling

Soil water samples were collected biweekly from Dec. 16^th^ 2008 to May 31^th^ 2010. A subsample of soil water was immediately stabilized for isotopic analyses (δ^13^C) of DOC (<1 µm) using mercury chloride (0.1% HgCl_2_ solution in 1∶113 or 1∶66 v/v). The samples were stored without headspace at 4°C until measurement. Volume, pH (Polylite Pro VP 120, Hamilton Messtechnik GmbH, Höchst-Forstel, Germany) and conductivity (TetraCon 325, WTW, Weilheim, Germany) of the remaining soil water were determined in the lab within 24 h. A subsample was used to measure the concentration of DOC and anions (Cl^−^, NO_3_^−^). The anion concentration was analyzed by ion chromatography (Dionex DX-500, Thermo Fischer Scientific, Idstein, Germany). The DOC concentration in the soil water was determined using a high-temperature total organic carbon analyzer (HighTOC II; Elementar Analysesysteme GmbH, Hanau, Germany).

Stable carbon isotope ratios (δ^13^C) of DOM were determined using the high performance liquid chromatography (HPLC) coupled online with the isotopic ratio mass spectrometry (IRMS). The system consisted of a ThermoFinnigan LC-IsoLink system (Thermo Electron, Bremen, Germany) coupled to a Delta+ XP isotope ratio mass spectrometer (Thermo Electron). The isotope ratios of DOM were measured on total carbon as the solution was free of carbonate, in consequence of the carbonate free loess cover [33] and the very low pH in the mineral soil (pH_KCl_ = 3.3 in 0–5 cm; [36]). Volumes of 25–50 µL soil solution were injected into the system without further pretreatments. Details about the HPLC-IRMS system, modifications and measurement procedures are described elsewhere [31], [39].

#### 2.5.2 Mineral soil sampling

Soil samples were collected at the end of the experiment after 539 days (May 31^st^, 2010). Two mineral soil cores (Ø 5 cm) from the middle of each mesocosm were transferred to the lab and stored at 4°C until further use. The cores were divided into 1 cm soil sections (0–1, 1–2, 2–3, 3–4 and 4–5 cm) and the two corresponding sections from each mesocosm were homogenized to get a composite sample. Soil was sieved (Ø 2 mm) and dried at 105°C for 24 h. Subsamples were ground in a ball mill (Retsch MM200, Haan, Germany). Organic C concentration was measured with an elemental analyzer (EA; vario Max, Elementar Analysesysteme GmbH, Hanau, Germany). Inorganic C was below the level of detection (LOD = 0.027% C) in all samples along the whole soil profiles (0–5 cm). To obtain isotope signatures (δ^13^C) of soil organic C, ground subsamples were weighed into tin capsules and measured using an EA-IRMS system [26]. Here, an EA (CE 1100) was coupled on-line via a Con Flo III interface with a Delta plus isotope ratio mass spectrometer (all supplied by Thermo Fisher, Bremen, Germany).

#### 2.5.3 Sampling of remaining leaf litter and O horizon

At the end of the experiment, the remaining leaf litter and O horizon on top of each mesocosm were collected. Samples were dried at 60°C until a constant weight was achieved. A subsample was ground in a mixer mill (Retsch MM2, Haan, Germany), dried (24 h at 105°C) and weighed into tin capsules. The C and N concentrations were measured using an automated C and N analyzer (Heraeus Elementar Vario EL, Hanau, Germany). To determine the isotope signature (δ^13^C) different EA-IRMS systems were used. For samples with a natural label an EA (NA1500 or NC2500) was coupled on-line via a Con Flo III with an IRMS Delta plus (Finnigan, MAT, Bremen, Germany). For enriched samples an EA NC1108 was coupled on-line via a Con Flo III interface with an IRMS Delta C (Finnigan, MAT, Bremen, Germany).

### 2.6 Calculations for the determination of litter-derived carbon

The stable carbon isotope ratios for isotopic measurements are reported in delta notation expressed in permil (Eq. 1).
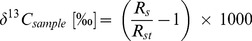
(1)where R_s_ is the ^13^C/^12^C ratio of the sample and R_st_ is the ratio of the international Vienna PeeDee Belemnite (V-PDB) standard. The measured δ^13^C values were corrected as described earlier [39], [40].

To calculate the fraction of litter derived C (*f*_litter_) in the different C pools (DOM, mineral soil, remaining litter and O horizon) we used a simple mixing model (Eq. 2; [41]).
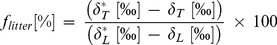
(2)where δ_T_* [‰] – δ_T_ [‰] is the difference between measured δ^13^C values of the C pool (DOM, mineral soil, remaining litter, O horizon) in a labeled treatment (δ_T_*) and under an equivalent unlabeled treatment (δ_T_) in the same block, δ_L_* [‰] – δ_L_ [‰] is the difference between measured δ^13^C values of the initial labeled (δ_L_*) beech or ash leaf litter and the equivalent unlabeled (δ_L_) leaf litter.

The determined litter derived C fraction (*f*_litter_) was used to calculate the percentage of litter-derived C (C_litter_) in the different C pools (Eq. 3).
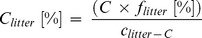
(3)where C is the determined carbon concentration in mg L^−1^ or mg g^−1^ multiplied with the sample volume in L (for DOM per sampling date) corrected for the mesocosm area or amount in g (for remaining litter and O horizon) or the carbon content in the soil calculated in g m^−2^ (for mineral soil), ƒ_litter_ [%] is the calculated fraction of litter-derived C, c_litter–C_ is the amount of litter-carbon in mg C (for remaining litter, O horizon and DOM) or g m^−2^ (for mineral soil).

### 2.7 Statistical analyses

Statistical analyses were done using SPSS (PAWS Statistics 18). To compare two different sampling dates or periods the non-parametric Wilcoxon signed-rank test (in case of no normal distribution of data) or the paired t-test (in case of normal distribution of data) was used. To calculate the litter-derived carbon in the different carbon pools (remaining leaf litter, O horizon, mineral soil and DOM) the unlabeled treatments (Be, BeAs, As) were used as references (Eq. 2) and therefore not included in the subsequent statistical analyses. To compare the different labeled treatments (Be*, Be*As, BeAs*, As*) a one-way ANOVA, followed by Tukey’s HSD post hoc test was used. To achieve normal distribution the data were log transformed. The litter parameters of the initial litter types were investigated using the Kruskal-Wallis test, followed by Mann-Whitney U test after determining a significant difference. A non-parametric Mann-Whitney U test was applied to compare two groups (e.g. pure vs. mixed treatments). The Spearman’s rank correlation coefficient (r_s_) was used to reveal a significant correlation between soil water conductivity and Cl^−^ or NO_3_^−^ concentrations. The significance level was set at p≤0.05.

## Results

### 3.1 Concentrations and isotope signatures of DOM in soil water

**Figure 1:**
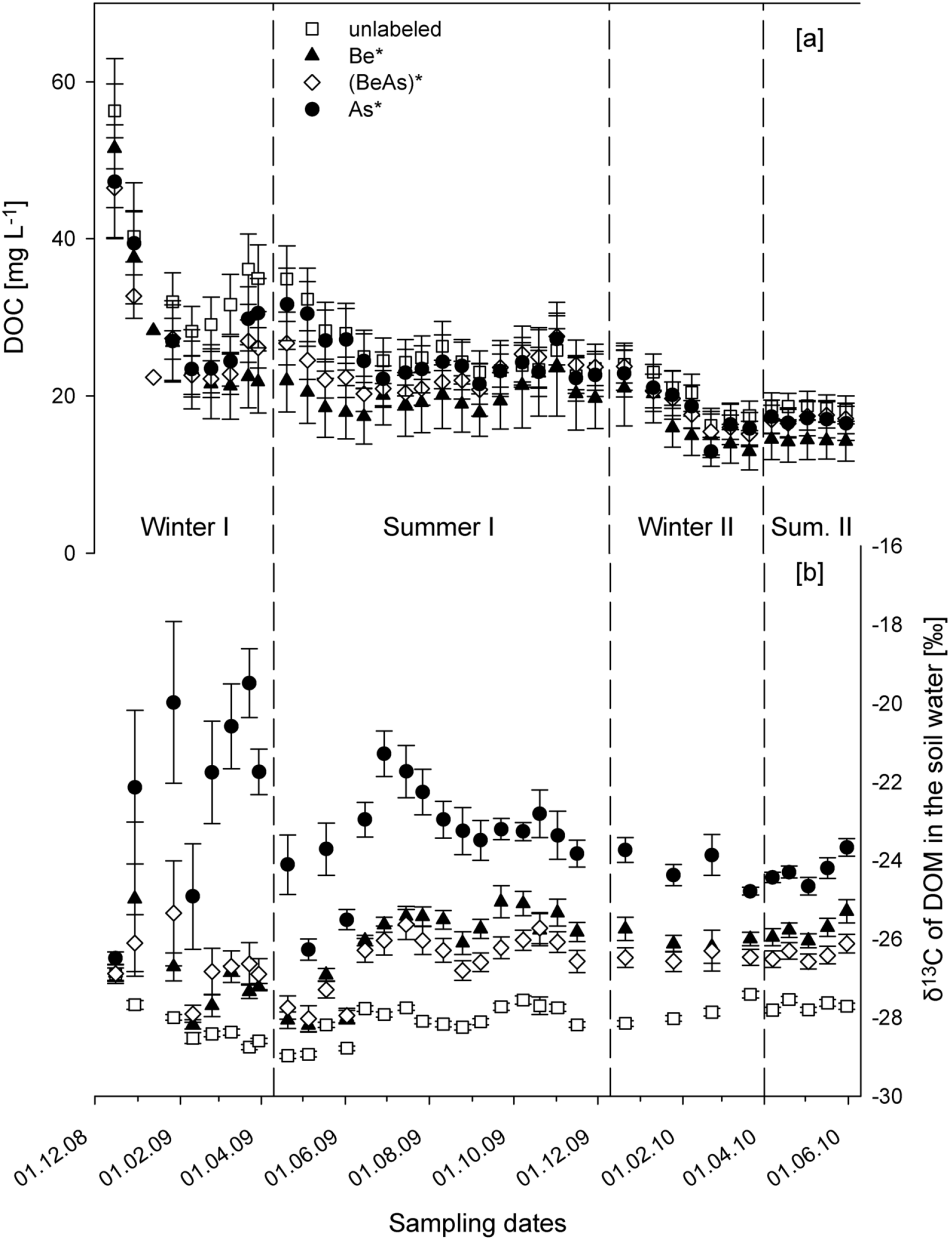
Measured DOM concentrations (1a) and δ^13^C values of the DOM (1b) in the soil water (mean ± standard error) under treatments with only labeled beech (Be*; n = 6), labeled ash (As*; n = 6), mixed ((BeAs)*; n = 12) and unlabeled litter treatments (unlabeled; n = 18). The dashed lines subdivide the experiment into the two winter (I: 16.12.08–30.03.09; II: 21.12.09–22.03.10) and two summer periods (I: 20.04.09–30.11.09; II: 07.04.10–31.05.10).

The DOC concentration in all treatments decreased during the whole experiment (Fig. 1a, S3 and S5 Tables). A significant (p<0.001, n = 33, paired t-test) decrease in the DOC concentration from 51.6 (±14.3 sd) mg C L^−1^ to 28.9±11.4 mg C L^−1^ occurred during the first two months of the experiment. During the experiment, we measured a first peak (27.2±13.5 mg C L^−1^) in the DOC concentration in all treatments from Mar. to Jun. 09 and a second smaller peak (26.1±11.7 mg C L^−1^) in Nov. 09. During both summer periods (I + II) we found higher average DOC concentrations in the soil water in ash (As, As*) treatments (25.0±11.5 mg C L^−1^) in comparison to beech (Be, Be*) treatments (18.5±8.5 mg C L^−1^) (Fig. 1a). Over the whole experiment, we also collected the highest cumulative amounts of DOC (367.6±134.0 mg C) in the soil water in the ash (As, As*) treatments and the lowest (265.6±117.8 mg C) in the beech (Be, Be*) treatments.

During the experiment, the δ^13^C values of the DOM in the unlabeled treatments (As, Be, BeAs) were nearly constant (−28.0±0.6 ‰; Fig. 1b, S3 and S5 Tables). Over the whole experimental period, the most enriched δ^13^C (−23.3±2.3 ‰) values were measured in the As* treatments in comparison to the mixed (−26.6±1.5 ‰) and Be* (−26.3±1.3 ‰) treatments. Only in the As* treatments we observed a first peak in the δ^13^C values (−22.1±3.6 ‰) during the first winter period and a second peak (−21.3±1.4 ‰) in the first summer period at the end of Jun. 09. In all treatments the highest variability between the six replicates occurred during the first winter period.

### 3.2 Partitioning of the litter-derived carbon into different carbon pools

#### 3.2.1 Litter-derived carbon in the soil water

**Figure 2:**
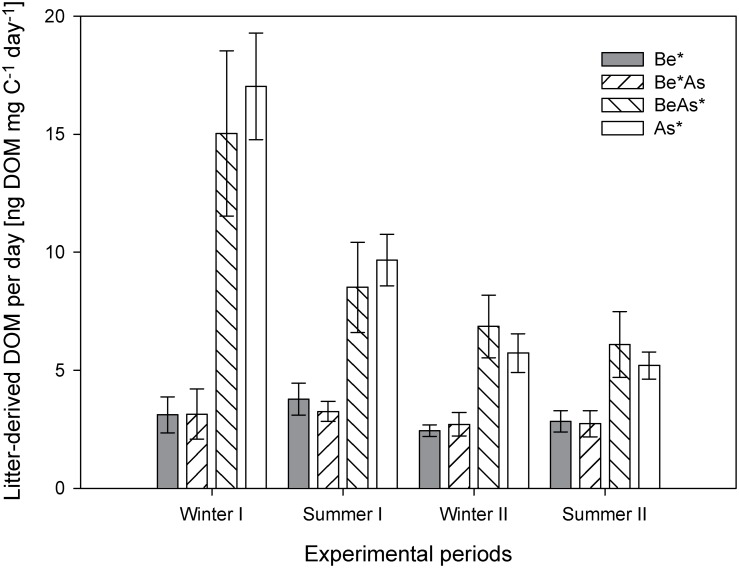
Calculated average daily release of litter-derived DOM (mean + standard error; n = 6) for the pure and mixed labeled (*) treatments of beech (Be) and ash (As) in the different winter (I: 16.12.08–30.03.09; II: 21.12.09–22.03.10) and summer periods (I: 20.04.09–30.11.09; II: 07.04.10–31.05.10).

**Figure 3:**
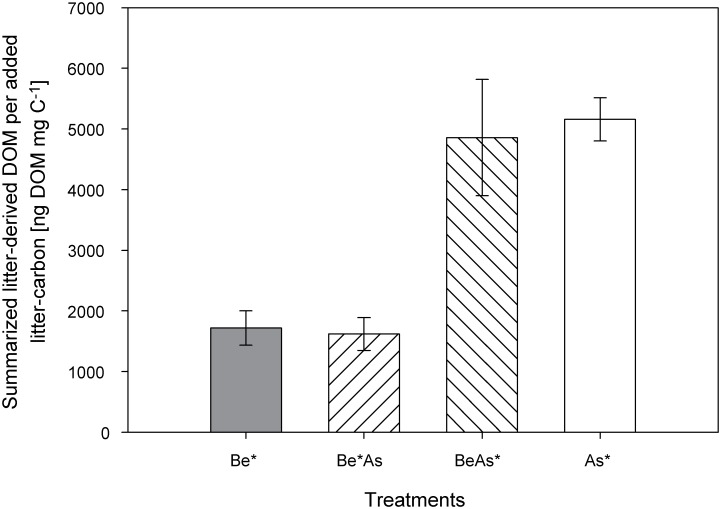
Determined amounts (± standard error) of litter-derived DOM per added litter-carbon summarized over the whole experiment for treatments with only labeled beech (Be*), labeled ash (As*) and mixed litter treatments (Be*As, BeAs*). The litter-derived DOM was significantly lower in the labeled beech (Be*, Be*As) treatments in comparison to the labeled ash (As*, BeAs*) treatments (p<0.01, n = 6, one-way ANOVA followed by Tukey’s HSD post hoc test).

Throughout the experiment, the average release of labeled litter derived carbon per day in the labeled ash (As*, BeAs*) treatments steadily decreased from 16.0±7.0 ng DOM mg C^−1^ day^−1^ in the first winter period to 5.6±0.7 ng DOM mg C^−1^ day^−1^ in the second summer period, whereas a nearly constant release (3.0±1.5 ng DOM mg C^−1^ day^−1^) was determined in the labeled beech (Be*, Be*As) treatments (Fig. 2). At the end of the experiment (Fig. 3) and for both summer periods (Fig. S3), significantly (p<0.01, n = 6, one-way ANOVA followed by Tukey’s HSD post hoc test) smaller amounts of litter-derived DOM were collected in the labeled beech (Be*, Be*As) treatments in comparison to labeled ash (As*, BeAs*) treatments (S4 Table). However, no significant differences were found between pure and mixed labeled beech (Be* vs. Be*As) and ash treatments (As* vs. BeAs*) neither for both summer periods (Fig. S3) nor at the end of the experiment (Fig. 3).

**Figure 4:**
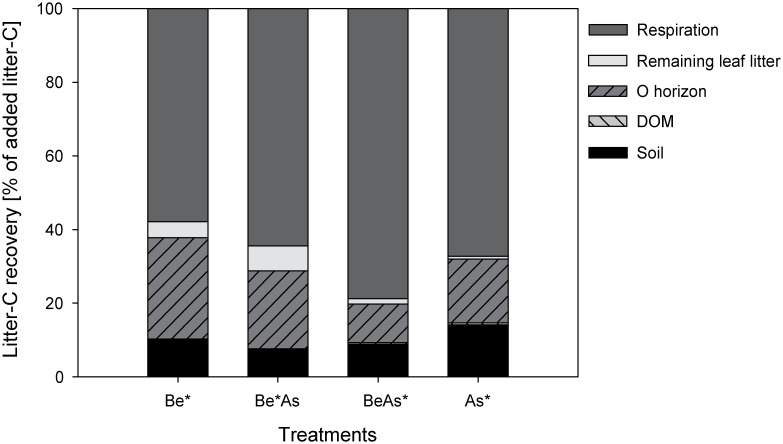
Average percent of litter-derived C (C_litter_) in the different carbon pools for the pure and mixed labeled (*) treatments of beech (Be) and ash (As) at the end of the experiment (n = 6). The DOM pool (<0.2%) for the labeled beech treatments in the diagram is too small to be visible. No significant (p>0.05, n = 6, one-way ANOVA) differences between pure and mixed labeled ash and beech treatments for litter-derived C in the remaining litter, the O horizon and for the mineral soil were found. For the litter-derived carbon in DOM we found significant (p<0.01, n = 6, one-way ANOVA) differences only between labeled ash (As*, BeAs*) and beech (Be*, Be*As) treatments (Fig. 3).

Surprisingly, the calculated amounts of litter-derived C in the soil water at the end of the experiment represented on average added leaf litter carbon of only 0.17±0.07% C_litter_ in labeled beech (Be*, Be*As) and 0.50±0.17% C_litter_ in labeled ash (As*, BeAs*) treatments (Fig. 4).

#### 3.2.2 Litter-derived carbon in the remaining leaf litter and O horizon

At the end of the experiment, we measured significantly higher amounts of remaining leaf litter in the pure labeled and unlabeled beech treatments (average 10.9±8.0%) in comparison to pure labeled and unlabeled ash (2.1±1.6%) treatments (p<0.01, n = 6, Mann-Whitney U test), and intermediate masses (9.3±7.7%) in mixed treatments (S4 Table). At the end of the experiment, we determined significantly lower (1.0±0.6%) remaining litter masses in the pure labeled (As*) in comparison to the pure unlabeled (As; 3.2±1.5%) ash treatments (p<0.01, n = 6, Mann-Whitney U test), whereas no significant differences in the remaining leaf litter were found between pure labeled (Be*) and unlabeled (Be) beech treatments. The remaining ash leaf litter mainly consisted of petioles. For both unlabeled litter types we found lower C/N ratios at the end of the experiment (Table 1). No differences or slightly increased C/N ratios were observed in the labeled beech and ash litter, respectively.

**Table 1:** Remaining added leaf litter mass [%] with determined C and N concentrations [mg g^−1^] and C/N ratios at the end of the experiment (31.05.2010) with the differences in comparison to the initial added leaf litter.

Remaining Leaf Litter							
	Mass [%]	C [mg g^−1^]		N [mg g^−1^]		C/N	
Litter type	31.05.2010	31.05.2010	Difference	31.05.2010	Difference	31.05.2010	Difference
**Be**	9.5 (2.8)^a^	419.2 (22.8)	88.1 (22.8)	14.1 (1.3)	−4.8 (1.8)	29.9 (3.7)^ac^	28.5 (3.8)
**As**	3.2 (1.5)^b^	430.4 (18.1)	56.4 (18.2)	13.1 (1.0)	−1.7 (1.1)	32.9 (2.5)^a^	9.7 (2.7)
Be*	12.3 (11.3)^a^	410.7 (44.3)	80.4 (44.6)	18.7 (3.8)	2.6 (3.8)	22.8 (5.8)^b^	0.3 (5.8)
As*	1.0 (0.6)^c^	390.2 (44.3)	65.8 (44.3)	16.0 (4.1)	3.9 (4.2)	25.2 (4.4)^cb^	−2.3 (4.5)

Values were calculated for all pure treatments of unlabeled and labeled (*) leaf litter of beech (Be) and ash (As). Represented are mean values with their standard deviation in parenthesis. High-letters represent significant differences (Kruskal-Wallis test followed by Mann-Whitney U test, p<0.05) between the different litter types.

On average, we determined labeled litter carbon (C_litter_) in the remaining litter layer of only 1.2 (±0.9) % in the labeled ash (As*, BeAs*) and 6.9 (±7.0) % in the labeled beech (Be*, Be*As) treatments at the end of the experiment (Fig. 4). In the O horizon, we also found lower litter-derived C (13.9±14.8% C_litter_) in the labeled ash (As*, BeAs*) in comparison to the labeled beech (Be*, Be*As; 24.4±17.7% C_litter_) treatments (Fig. 4). However, we found no significant (p>0.05, n = 6, one-way ANOVA) differences in litter-derived C between pure and mixed labeled ash and beech treatments, neither in the remaining litter nor in the O horizon at the end of the experiment.

#### 3.2.3 Litter-derived carbon in the mineral soil

For the carbon content in the mineral soil we observed on average a strong decrease from 12.3 (±6.5) % C in 0–1 cm to 2.0 (±0.7) % C in 4–5 cm depth in all treatments (S4 Table). We found significantly (p = 0.04, n = 12, Mann-Whitney U test) higher δ^13^C values (−24.1±3.9 ‰) in the labeled pure (Be*, As*) treatments in comparison to the mixed treatments (−26.8±1.0 ‰) in the 0–1 cm depth layer. However, in a depth of 4–5 cm the δ^13^C values in all labeled treatments (−27.7±0.4 ‰) were in the range of the unlabeled treatments (−27.8±0.4 ‰), which were homogenous for all depths (0–5 cm).

In the labeled beech treatments, we determined an average recovery of litter-derived C of 10.1 (±13.3) % C_litter_ (Be*) or 7.4 (±6.5) % C_litter_ (Be*As) and slightly higher values in labeled ash treatments with 14.1 (±5.8) % C_litter_ (As*) or 8.8 (±9.4) % C_litter_ (BeAs*) in the 0–5 cm of the mineral soil (Fig. 4). In all treatments, most of this litter-derived C (on average 78.4±24.2%) was already located in 0–2 cm soil depth. However, we found no significant differences (p>0.05, n = 6, one-way ANOVA) between pure and mixed labeled ash and beech treatments for the mineral soil. In general, the mineral soil together with the O horizon held in total around 30% (29.2±19.1%) of litter-derived carbon in all treatments.

### 3.3 Inorganic water chemistry

With cumulative amounts of 13.6 (±1.1) L soil water we found no significant differences (p>0.05, n = 6, one-way ANOVA) between the treatments at the end of the experiment (S3 Table). The collected volumes were in agreement with the annual precipitation. After a significant (p<0.001, n = 33, Wilcoxon signed-rank test) decrease in all treatments from 6.5 (±0.4) to 4.6 (±0.3) during the first two months, the soil water pH was around 4.2 (±0.2) in all treatments during the rest of the experiment (S3 Table).

Among the anions, nitrate (NO_3_^−^) occurred in the highest concentrations in soil water. For the winter periods (Dec. – Mar.), we measured on average 37.4 (±18.6) mg NO_3_^−^ L^−1^ for all treatments, whereas they clearly increased in two steps up to 120.4 (±35.5) and 135.5 (±67.5) mg NO_3_^−^ L^−1^ during the first summer period (S3 Table). The average soil water Cl^−^ concentration for all treatments was 1.7 (±0.9) mg Cl^−^ L^−1^, except for the first winter period (S3 Table). Here, we revealed a trend to higher Cl^−^ concentrations in ash (As, As*) treatments (18.7±18.1 mg Cl^−^ L^−1^) compared to beech (Be, Be*) treatments (3.7±1.7 mg Cl^−^ L^−1^).

**Figure 5:**
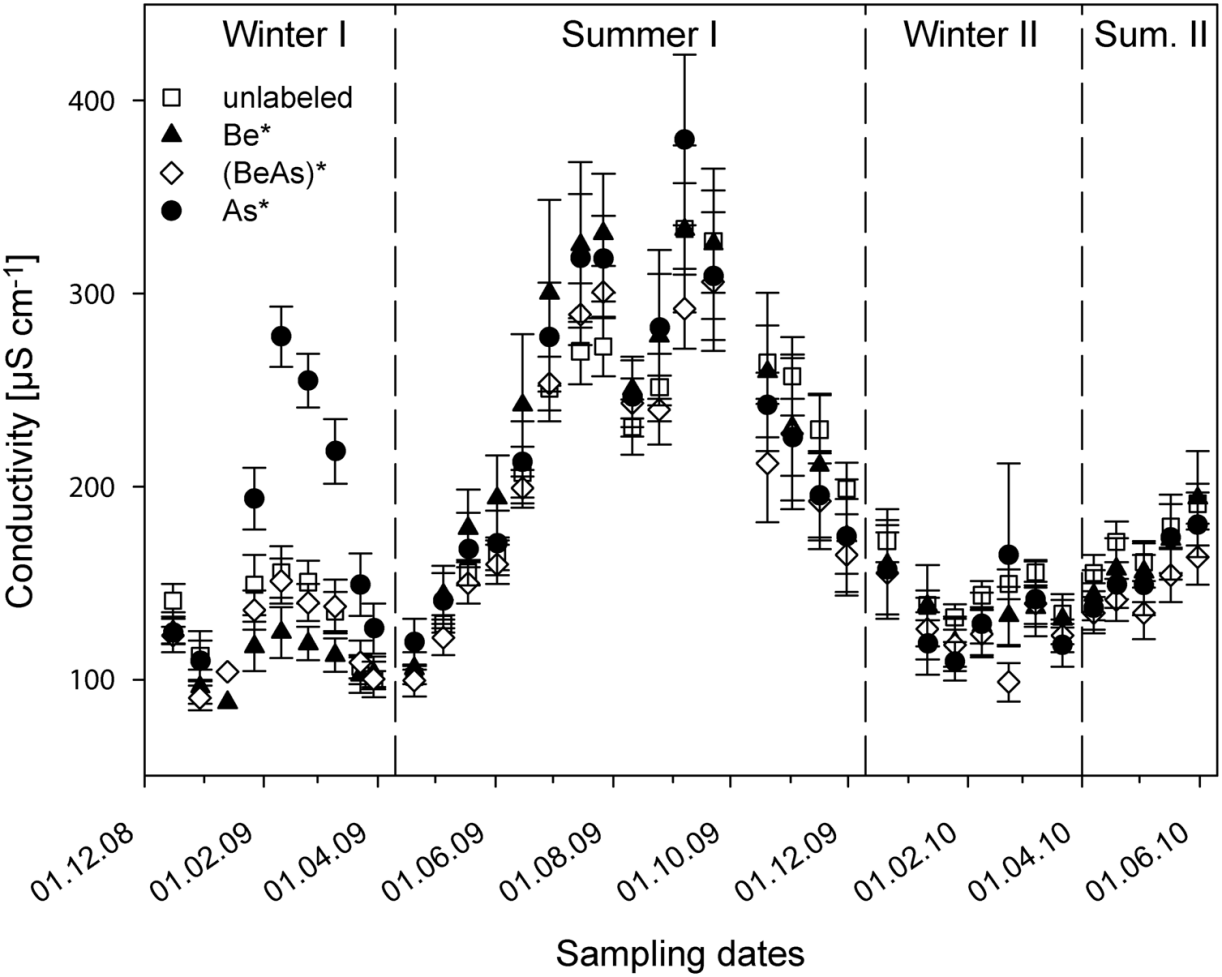
Measured conductivities (mean values ± standard error) in soil water under treatments with only labeled beech (Be*; n = 6), labeled ash (As*; n = 6), mixed ((BeAs)*; n = 12) and unlabeled litter treatments (unlabeled; n = 18). The dashed lines subdivide the experiment into the two winter (I: 16.12.08–30.03.09; II: 21.12.09–22.03.10) and two summer periods (I: 20.04.09–30.11.09; II: 07.04.10–31.05.10).

The soil water conductivity showed two distinct peaks (Fig. 5, S3 Table). During the first winter period (Dec. 08 – Mar. 09), we determined the best correlation (r_s_ = 0.710, p<0.01) between conductivity and Cl^−^ concentrations (Fig. S4), whereas for both summer periods the conductivity showed a very high correlation (r_s_ = 0.981, p<0.01) with the measured NO_3_^−^ concentrations (Fig. S5).

## Discussion

### 4.1 Effects of litter quality on DOM release

With respect to our first hypothesis, we revealed that the export of litter-derived DOM increases with higher leaf litter decomposability.

In earlier studies, the C/N ratios of leaf litter were often found to explain the decomposability of a specific litter type [22], [23]. In our experiment, the C/N ratios of the labeled beech and ash leaf litter were lower due to the labeling in comparison to the unlabeled beech and ash leaf litter (S1 Table). At the end of the experiment, we determined no differences between the pure beech (Be*, Be) treatments in the remaining leaf litter masses, whereas significantly lower litter masses were found in the pure labeled ash (As*) treatments in comparison to the unlabeled (As) treatments. This indicates that the C/N ratio did not influence the decomposition of beech litter, but that a lower C/N ration slightly increased the decomposition in the labeled ash treatments. Therefore, our results might not exactly represent the decomposition processes and release of litter-derived DOM of the native (unlabeled) beech and ash leaf litter at the study site. However, we observed that the lower C/N ratios in the labeled leaf litter types (As*, Be*) did not change the overall decomposition pattern as we determined for both pure labeled and unlabeled ash treatments (As*, As) significantly lower remaining litter masses in comparison to the respective pure labeled and unlabeled beech treatments (Be*, Be). Therefore, our results still allow conclusions on the effect of litter decomposability on the release of litter-derived DOM and the formation of SOM. This also indicates that in our experiment the litter decomposability was probably further influenced by the Lignin/N ratio, water or cation content, cuticula constituents or secondary metabolites like tannins or other phenolic compounds of the leaf litter types [17], [23], [42], [43].

In accordance to the literature [43], ash leaf litter in comparison to beech leaf litter increased the release of litter-derived carbon in DOM, which can be transferred into soil horizons deeper than 5 cm mineral soil, due to a higher litter quality or decomposability. The differences in the litter decomposability between ash and beech induced different release patterns of litter-derived DOM (Fig. 2). As also reported for other leaf litter types [43], [44], in the labeled ash treatments (As*, BeAs*) we observed the highest release of litter-derived DOM per day at the beginning followed by an exponential decrease throughout the experiment. This indicates that the litter decomposition stage is of high importance for the decomposition of ash leaf litter. Most of the litter-derived DOM was directly released in the first winter period (winter I) probably due to the physical destruction of the added leaf litter and hydraulic leaching of litter components caused by freeze-thawing cycles [45]. It is known that in winter under high moisture condition (e.g. snowmelt) and when microbial activity and decomposition are lower the chemical composition of DOM shows the highest plant-derived carbon and includes high nutrient contents of fresh disrupted microbial biomass or other easily decomposable soluble organic matter [12], [20], [43], [46]. For the first winter, we found a good correlation between the conductivity and the Cl^−^ (S4 Figure). The Cl^−^ as a constituent part of the liquid phase in plant cells can be easily leached from the added leaf litter by precipitation or snowmelt [47]. During the second winter, we did not detect a correlation between conductivity and Cl^−^ concentrations probably because most Cl^−^ was already leached. For both summer periods, we found a good correlation between conductivity and NO_3_^−^ (S5 Figure) that we considered as a result of soil fauna (especially microbial) decomposition activities. However, for beech leaf litter the decomposition stage seems to be of less importance for the release of litter-derived DOM. In comparison to a former study [43], we observed a nearly constant release of litter-derived DOM per day in the labeled beech treatments (Be*, Be*As) with minimal fluctuations between the winter and summer periods (Fig. 2). We conclude that the leaf litter decomposability, including the structural and chemical characteristics of the litter, is an important factor influencing the intensity and time frame of the litter-derived DOM release.

### 4.2 Litter mixture effects on DOM release

Unexpectedly in contrast to our second hypothesis, we did not observe an influencing effect of the litter mixture on the release of litter-derived DOM. We expected a higher export of litter-derived DOM in BeAs* and lower in Be*As compared to the pure (As*, Be*) treatments due to a selective decomposition of ash litter (of higher litter decomposability) by the soil fauna (micro-, meso-, makro-). However, we found no significant differences in the release of litter-derived DOM neither between the pure and mixed labeled treatments of ash nor of beech (Figs. 2 and 3 and S3 Figure). An influencing effect of the litter mixture on the decomposition of the individual leaf litter (beech and ash) was also not detectable in the remaining (labeled) leaf litter, O horizon and mineral soil (0–5 cm) at the end of the experiment (Fig. 4).

Soil fauna can enhance the litter decomposition due to bioturbation and the breakup of litter material by their feeding activities and also increase the accessibility of food sources to microorganisms [48], [49]. Using gauze (on top and at the bottom of the mesocosms) we prevented soil fauna (meso- and macro-) to enter the mesocosms, but we did not exclude the present soil fauna by taking intact soil cores at the beginning of the experiment. However, in a companion study of this experiment it was found that only three of eleven primary decomposer species (taxa from Oribatida, Collembola, and Diplopoda) were significantly influenced by the mixed litter [50]. Furthermore, no significant differences in the release of litter-derived CO_2_ emissions were found between the various treatments from May 7^th^ 09 [36] underlining that the microbial organisms can only decompose organic material, which they can access [29], [51]. Feinstein and Blackwood [52], [53] already showed for a mixed deciduous forest that the fungal community composition on individual leaves was only slightly affected be the litter type, whereas the habitat and site conditions explain most of the variability in the fungal community. Accordingly, our results demonstrate that, even if the soil fauna (micro-, meso-, macro-) influences the decomposition processes of a specific litter type (due to variable preferences) in our experiment, they were not strong enough to be mirrored in the release and transport of litter-derived DOM into mineral soil horizons deeper than 5 cm. Obviously, our long observation time leads to a harmonization of short term effects.

### 4.3 Leaf litter contribution to the SOM formation and C-cycle

With our experiment, we revealed that litter-derived DOM represented only a minor part of the whole DOM flux in 5 cm mineral soil depth. Our results clearly illustrate that nearly all (∼99%) soil water DOM in 5 cm mineral soil depth originated from an “old” SOM pool, which is in line with former studies [20], [26], [54], [55]. Independent of the litter decomposability and litter mixture only a minor part of DOM was litter-derived C with less than 1% C_litter_ in all labeled treatments.

In general, we recovered around 30% of labeled litter-derived carbon in the whole soil horizons (organic and mineral) in the different mesocosms at the end of the experiment (Fig. 4). These percentages of recovered total litter-derived C at the end of the experiment suggest that on average 67.3±21.7% C_litter_ was lost in all treatments, probably as CO_2_ into the atmosphere due to soil fauna (micro-, meso-, macro-) respiration, which is in line with results from the literature [20], [55], [56]. In labeled beech treatments, most of the litter-derived carbon was found in the O horizon, whereas it was nearly equally distributed between the O horizon and the mineral soil in the labeled ash treatments (Fig. 4). This indicates that higher litter decomposability slightly increased the vertical carbon transfer and SOM formation. However, for the mineral soil around 80% of the recovered litter-derived carbon was already localized in the first 2 cm. These results underline the large retention of litter-derived carbon in the upper organic and mineral soil horizons.

## Conclusion

We found that ash litter with lower lignin content was decomposed faster than beech litter as the export of litter-derived carbon in the soil solution was higher in treatments with higher decomposable (ash) litter. This clearly indicates an enhanced transfer of litter-derived DOM into soil horizons deeper than 5 cm mineral soil depth with the addition of high-quality leaf litter. Surprisingly, the contribution of litter-derived carbon into the DOM pool was very low. We found less than 1% of litter-derived carbon (0.17±0.07% for beech and 0.50±0.17% for ash) in the soil DOM pool at the end of the experiment. This implies that more than 99% of carbon in the forest soil DOM pool originates from an “old” SOM pool. We localized around 30% of litter-derived carbon in the upper organic and mineral soil horizons (until 5 cm mineral soil depth) at the end of the experiment, which underlines the strong potential of soil to retain carbon.

Most interestingly, we could not detect any differences in labeled ash or beech litter between pure and mixed treatments. This indicates that the release of litter-derived DOM and the formation of litter-derived SOM were not significantly influenced by a preferential decomposition of ash litter (faster decomposable) in mixture with beech litter by the soil fauna (micro-, meso-, macro-).

Overall our results suggest that 1) litter derived carbon is of low importance for the DOM formation and carbon loss with soil water and 2) the mixture of leaf litter has no or only minor effects on the release of litter-derived DOM and the formation of new SOM.

## Supporting Information

S1 Figure Experimental setup at the study site with mesocosms arranged in three blocks and two mesocosms of the following treatments at each block: 1) unlabeled beech litter (Be), 2) 1∶1 (m/m) mixture of unlabeled beech and ash litter (BeAs), 3) unlabeled ash litter (As), 4) labeled beech litter (Be*), 5) 1∶1 (m/m) of labeled beech and unlabeled ash litter (Be*As), 6) 1∶1 (m/m) of unlabeled beech and labeled ash litter (BeAs*), 7) labeled ash litter (As*).
(TIF)

S2 Figure **Environmental parameters collected at a tall tower located in the Weberstedter Holz of the Hainich National Park.** Mean values (± sd) for soil moisture (SM, n = 4) and soil temperature (ST, n = 2) for the time frame between two sampling points are represented. For precipitation all collected volumes between two sampling points were summarized. The dashed lines subdivide the experiment into the two winter (I: 16.12.08–30.03.09; II: 21.12.09–22.03.10) and two summer periods (I: 20.04.09–30.11.09; II: 07.04.10–31.05.10).
(TIF)

S3 Figure Determined amounts (± standard error) of litter-derived DOM per added litter-carbon for treatments with only labeled beech (Be*), labeled ash (As*) and mixed litter treatments (Be*As, BeAs*) summarized over both summer periods (20.04. – 30.11.09 and 07.04. – 31.05.10). The litter-derived DOM was significantly lower in the labeled beech (Be*, Be*As) treatments in comparison to the labeled ash (As*, BeAs*) treatments (p<0.01, n = 6, one-way ANOVA followed by Tukey’s HSD post hoc test).
(TIF)

S4 Figure **Correlation between conductivity and Cl^−^ for all treatments in the time of the first winter period (10.02.09–30.03.09; n = 204) represented by Spearman’s rank correlation coefficient (rho) and linear regression with 95% confidence interval (blue lines).**
(TIF)

S5 Figure **Correlation between conductivity and NO_3_^−^ for all treatments and both summer periods (20.04. – 30.11.09 and 07.04. – 31.05.10, n = 874) represented by Spearman’s rank correlation coefficient (rho) and linear regression with 95% confidence interval (blue lines).**
(TIF)

S1 Table Isotopic signature (δ^13^C) and litter quality parameters (mean values with standard deviation in parenthesis) for the unlabeled (n = 4) and labeled (*; n = 12) leaf litter of beech (Be) and ash (As) at the beginning of the experiment (for lignin n = 4) (data from Langenbruch et al. 2013). High-letters represent significant differences (Kruskal-Wallis test followed by Mann-Whitney U test, p<0.05) between the different litter types.
(DOC)

S2 Table **Collected environmental data (mean values ± sd) for soil moisture (SM, n = 4) and soil temperature (ST, n = 2) for the time frame between two sampling points are represented.** For precipitation all collected volumes between two sampling points were summarized.
(XLS)

S3 Table **Measured values for soil water volume, isotope signature (δ^13^C) and concentration of DOM, pH, conductivity and anion concentration (Cl^−^, NO_3_^−^) as well as the calculated amount of litter-derived DOM at the individual sampling points.**
(XLS)

S4 Table **Recovery of litter-derived carbon calculated for the different carbon pools (remaining leaf litter, O horizon, mineral soil, DOM and respiration) at the end of the experiment.**
(XLS)

S5 Table **Mean values with lower and higher confidence interval for the measured DOM concentration, δ^13^C and conductivity values determined in the soil water.**
(XLSX)
