# Supplementary figures and images for: Influence of Litter Diversity on Dissolved Organic Matter Release and Soil Carbon Formation in a Mixed Beech Forest
Source: PLoS One. 2014 Dec 8;9(12):e114040. doi: 10.1371/journal.pone.0114040 (PMC4259385; doi:10.1371/journal.pone.0114040)

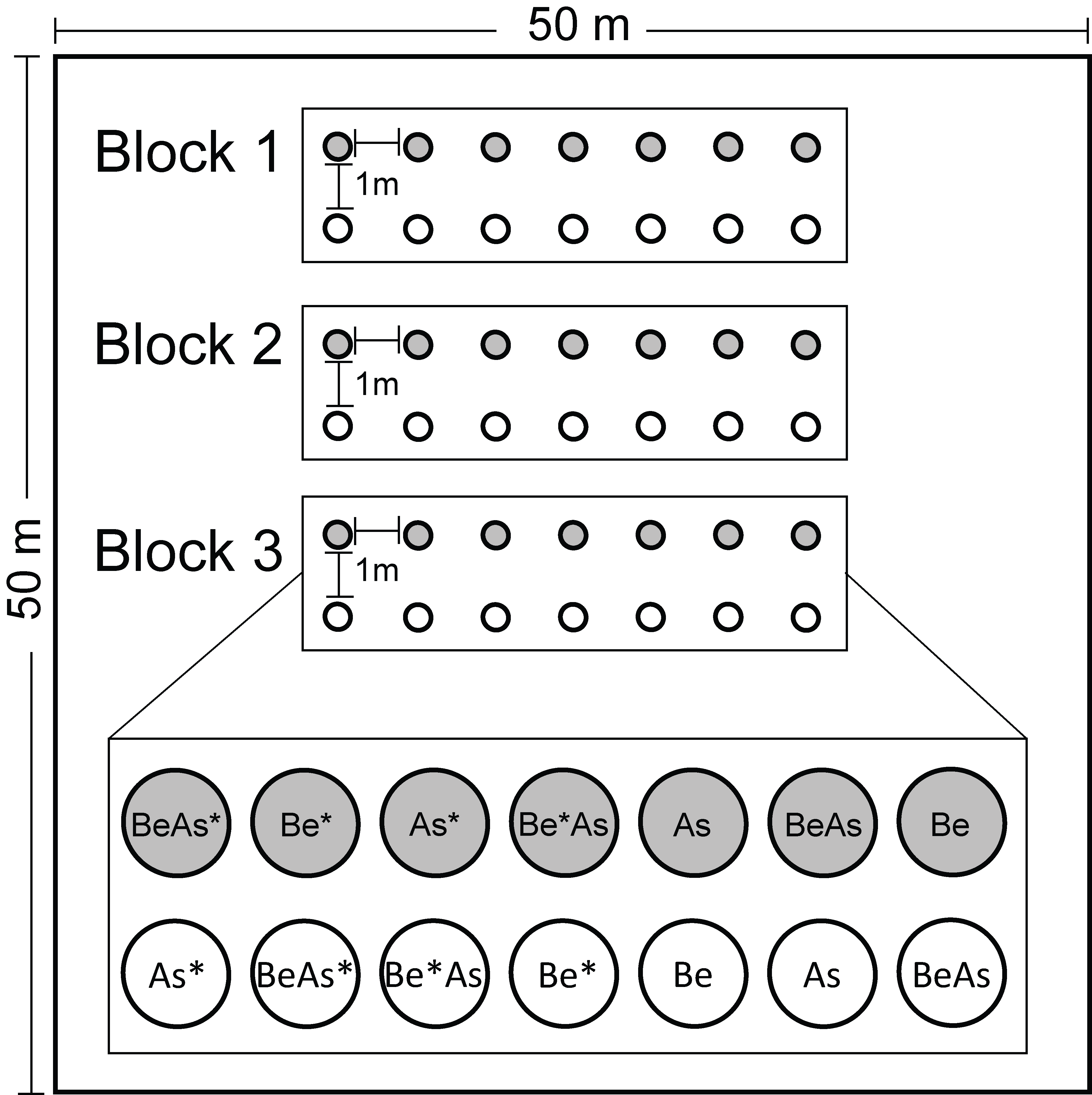

Supplement: S1 Figure — Experimental setup at the study site with mesocosms arranged in three blocks and two mesocosms of the following treatments at each block: 1) unlabeled beech litter (Be), 2) 1∶1 (m/m) mixture of unlabeled beech and ash litter (BeAs), 3) unlabeled ash litter (As), 4) labeled beech litter (Be*), 5) 1∶1 (m/m) of labeled beech and unlabeled ash litter (Be*As), 6) 1∶1 (m/m) of unlabeled beech and labeled ash litter (BeAs*), 7) labeled ash litter (As*). (TIF) [file pone.0114040.s001.tif]

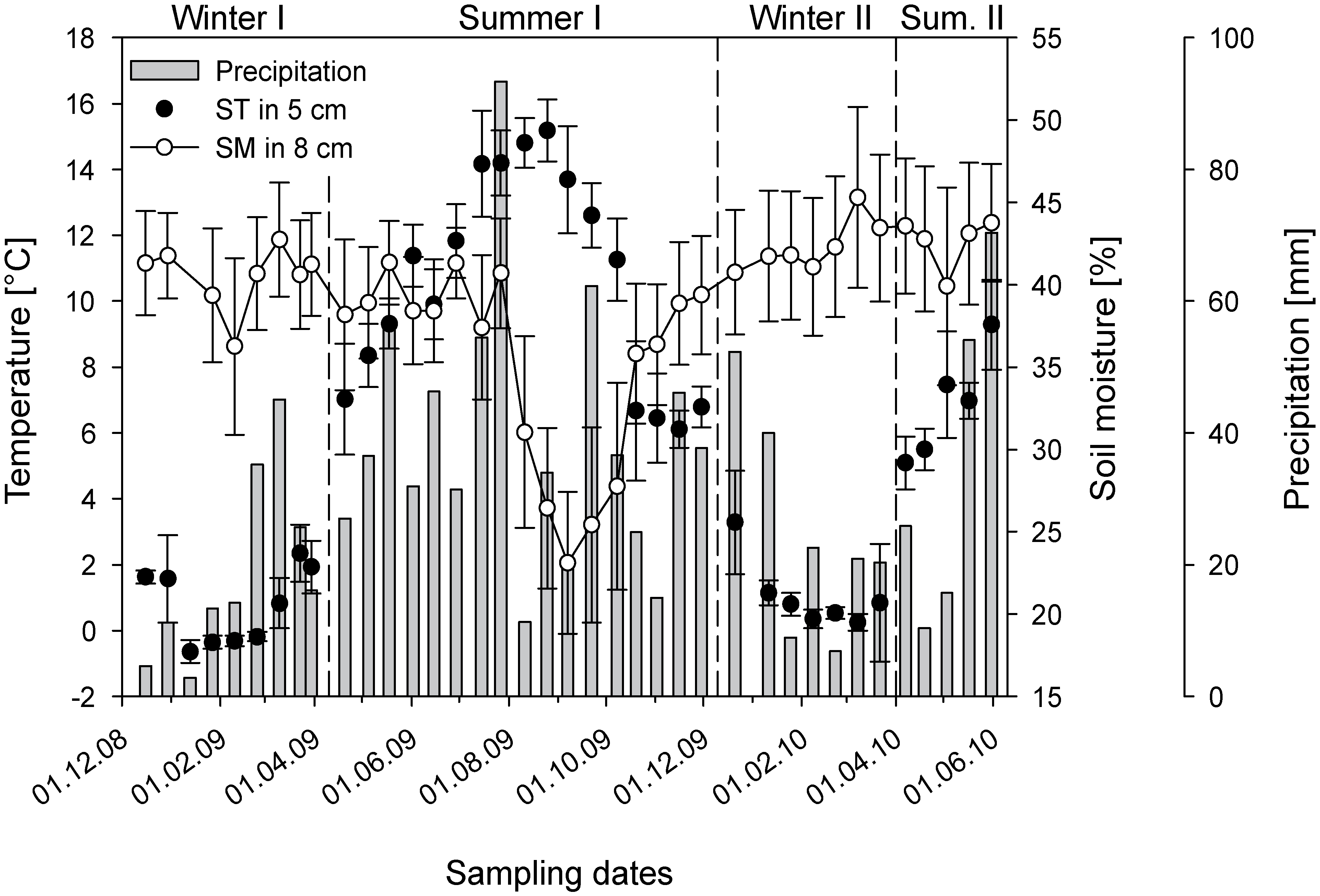

Supplement: S2 Figure — Environmental parameters collected at a tall tower located in the Weberstedter Holz of the Hainich National Park. Mean values (± sd) for soil moisture (SM, n = 4) and soil temperature (ST, n = 2) for the time frame between two sampling points are represented. For precipitation all collected volumes between two sampling points were summarized. The dashed lines subdivide the experiment into the two winter (I: 16.12.08–30.03.09; II: 21.12.09–22.03.10) and two summer periods (I: 20.04.09–30.11.09; II: 07.04.10–31.05.10). (TIF) [file pone.0114040.s002.tif]

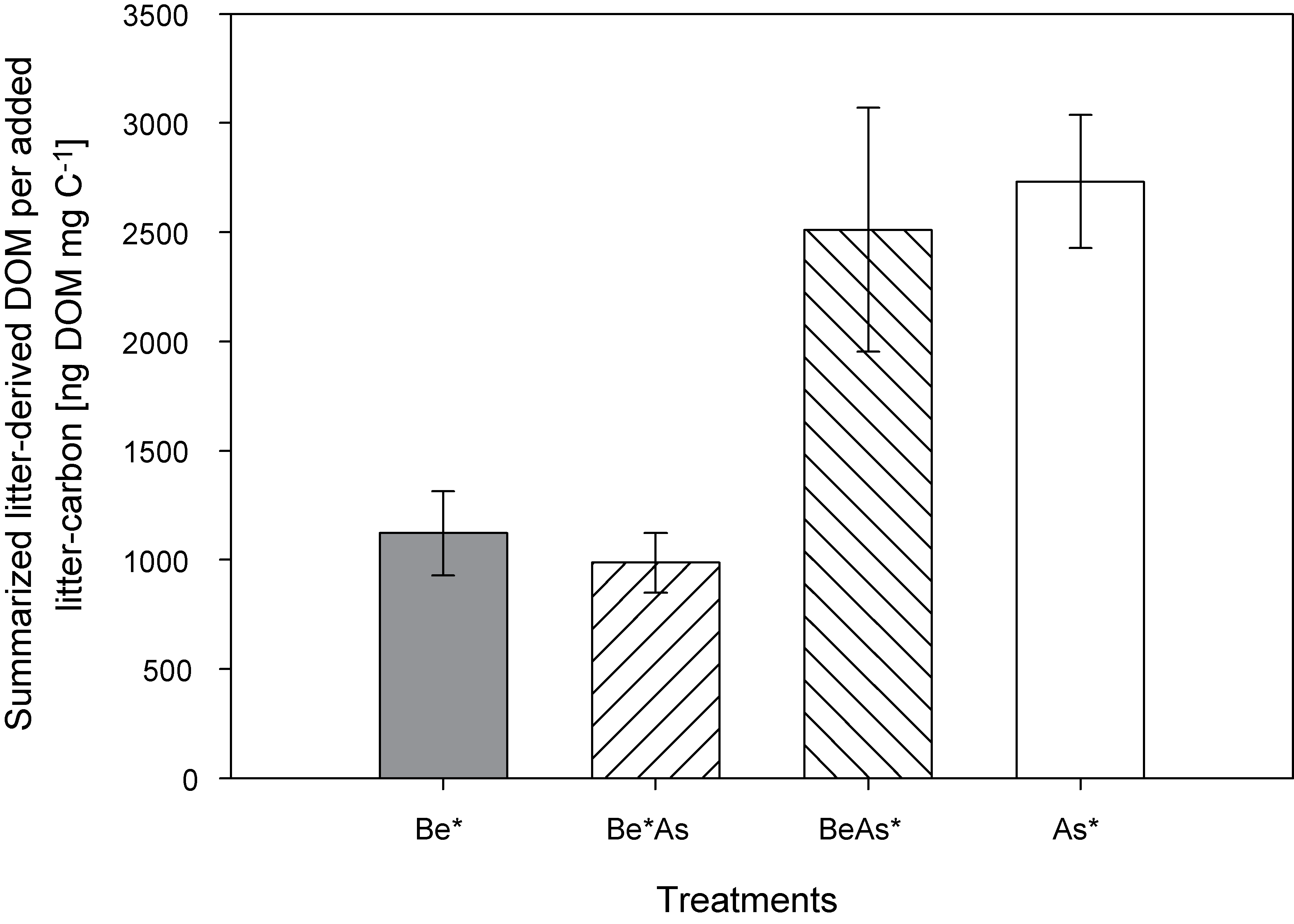

Supplement: S3 Figure — Determined amounts (± standard error) of litter-derived DOM per added litter-carbon for treatments with only labeled beech (Be*), labeled ash (As*) and mixed litter treatments (Be*As, BeAs*) summarized over both summer periods (20.04. – 30.11.09 and 07.04. – 31.05.10). The litter-derived DOM was significantly lower in the labeled beech (Be*, Be*As) treatments in comparison to the labeled ash (As*, BeAs*) treatments (p<0.01, n = 6, one-way ANOVA followed by Tukey’s HSD post hoc test). (TIF) [file pone.0114040.s003.tif]

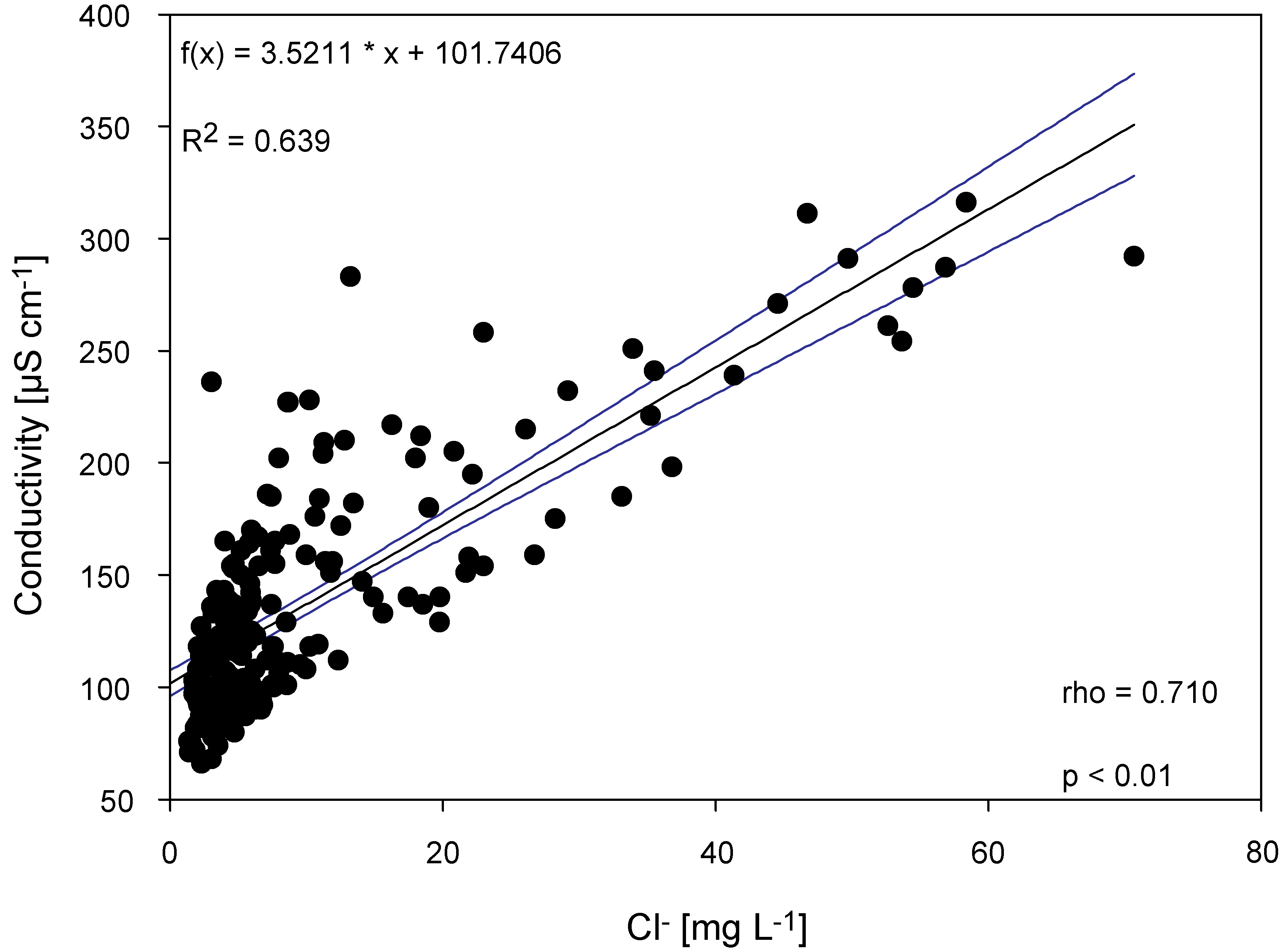

Supplement: S4 Figure — Correlation between conductivity and Cl− for all treatments in the time of the first winter period (10.02.09–30.03.09; n = 204) represented by Spearman’s rank correlation coefficient (rho) and linear regression with 95% confidence interval (blue lines). (TIF) [file pone.0114040.s004.tif]

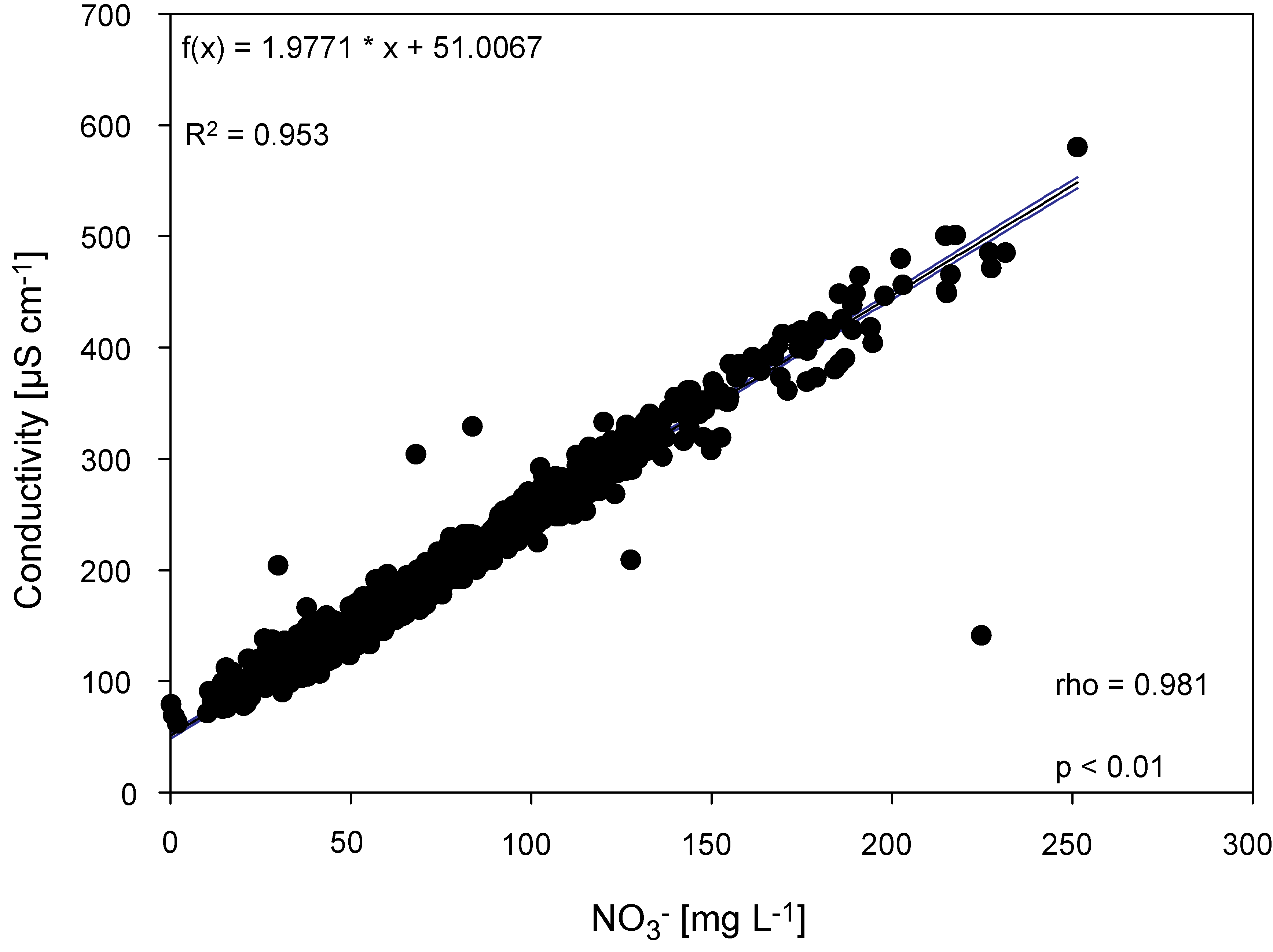

Supplement: S5 Figure — Correlation between conductivity and NO3− for all treatments and both summer periods (20.04. – 30.11.09 and 07.04. – 31.05.10, n = 874) represented by Spearman’s rank correlation coefficient (rho) and linear regression with 95% confidence interval (blue lines). (TIF) [file pone.0114040.s005.tif]
