# Supplementary material for: Influence of Litter Diversity on Dissolved Organic Matter Release and Soil Carbon Formation in a Mixed Beech Forest
Source: PLoS One. 2014 Dec 8;9(12):e114040. doi: 10.1371/journal.pone.0114040 (PMC4259385; doi:10.1371/journal.pone.0114040)
Supplement: S1 Table — Isotopic signature (δ13C) and litter quality parameters (mean values with standard deviation in parenthesis) for the unlabeled (n = 4) and labeled (*; n = 12) leaf litter of beech (Be) and ash (As) at the beginning of the experiment (for lignin n = 4) (data from Langenbruch et al. 2013). High-letters represent significant differences (Kruskal-Wallis test followed by Mann-Whitney U test, p<0.05) between the different litter types. (DOC) [file pone.0114040.s006.doc]

**Table S1** Isotopic signature (δ^13^C) and litter quality parameters (mean values with standard deviation in parenthesis) for the unlabeled (n = 4) and labeled (*; n = 12) leaf litter of beech (Be) and ash (As) at the beginning of the experiment (for lignin n = 4) (data from [36]). High-letters represent significant differences (Kruskal-Wallis test followed by Mann-Whitney U test, p < 0.05) between the different litter types.

|  | **Litter quality parameter** | | | | |
| --- | --- | --- | --- | --- | --- |
| **Litter** | **δ^13^C [‰]** | **C [mg g^-1^]** | **N [mg g^-1^]** | **C/N** | **Lignin [mg g^-1^]** |
| **Be** | -29.3 (0.3)^a^ | 507.3 (0.9)^a^ | 9.3 (1.3)^a^ | 58.5 (0.8)^a^ | 301.1 (3.9)^a^ |
| **As** | -28.1 (0.6)^a^ | 486.7 (1.0)^b^ | 11.4 (0.3)^a^ | 42.6 (1.1)^a^ | 200.6 (2.4)^b^ |
| **Be*** | 118.1 (1.7)^b^ | 492.3 (1.3)^c^ | 21.3 (0.4)^b^ | 23.1 (0.4)^b^ | 240.6 (4.4)^c^ |
| **As*** | 155.0 (5.2)^c^ | 456.1 (1.9)^d^ | 19.9 (0.9)^c^ | 22.9 (1.1)^b^ | 177.5 (2.1)^d^ |
